
JOURNAL INFORMATION
==============================
NLM Title Abbreviation: Alcohol Res Health
Journal ID: Alcohol Res Health
NLM Journal ID: 100900708
Journal ID: ARH

Alcohol Research & Health

ISSN: 1535-7414
EISSN: 1930-0573
Publisher: National Institute on Alcohol Abuse and Alcoholism

ARTICLE INFORMATION
==============================
PMCID: PMC3860453
Article ID: arh-31-2-177
Article version: 1
Subjects: Glossary

Glossary

Print publication date: 2008
Volume: 31
Issue: 2
First page: 177
Last page: 179
Copyright year: 2008
License: Unless otherwise noted in the text, all material appearing in this journal is in the public domain and may be reproduced without permission. Citation of the source is appreciated.
License URL: https://creativecommons.org/publicdomain/mark/1.0/

Comment in: The Genetics of Alcohol and Other Drug Dependence 31 2 2008 111 118 Alcohol Research & Health PMC3860452 23584813
Comment in: Shared Mechanisms of Alcohol and Other Drugs 31 2 2008 137 147 Alcohol Research & Health PMC3860454 23584815
Comment in: The Influence of Stress on the Transition From Drug Use to Addiction 31 2 2008 119 136 Alcohol Research & Health PMC3860459 23584814

==============================
γ-Aminobutyric acid (GABA): A neurotransmitter that in the brain acts to reduce the activity of the signal-receiving nerve cell (i.e., an inhibitory neurotransmitter); helps to restore homeostasis after a stressful situation.

Adoption study: Type of behavioral genetic study that compares the characteristics of adopted individuals with those of their biological and adoptive families in order to determine the relative contributions of genetic and environmental factors to a given characteristic. If there is greater similarity between the adoptee and the biological family than between the adoptee and the adoptive family, genetic factors are thought to play an important role in the characteristic under investigation.

Adrenal glands: Small structures located on top of the kidneys comprising an outer layer (i.e., cortex) and an inner layer (i.e., medulla) that produce numerous hormones. The primary adrenal hormones are the glucocorticoids, which are produced in the cortex. The inner layer of the adrenal glands also produces two substances—epinephrine (adrenaline) and norepinephrine (noradrenaline)—released as part of the fight-or-flight response to various stressors.

Agonist: A substance that binds to a specific receptor and triggers a response in the cell. It mimics the action of a compound that binds to the same receptor.

Allele: One of two or more variants of a gene or other DNA sequences. Different alleles of a gene generally serve the same function (e.g., code for a protein that affects eye color) but may produce different phenotypes (e.g., blue eyes or brown eyes). Some alleles may be defective and produce a protein that has no function or an abnormal function.

Allostasis: The process of achieving stability, or homeostasis, in response to environmental changes (e.g., exposure to stressors) through physiological or behavioral change (e.g., changes in the HPA axis, the autonomic nervous system, or signaling molecules [i.e., cytokines], or other systems). These alterations generally lead to short-term adaption to predictable and unpredictable events.

Allostatic load: The cumulative physiological cost to the body of chronic exposure to the stress response and allostasis.

Amygdala: Almond-shaped groups of nerve cells (i.e., neurons) located deep in the brain that are involved in the processing and memory of emotional reactions. The amygdala are part of the limbic system.

Anxiolysis: Relief of the symptoms of anxiety.

Antagonist: A substance that prevents or reverses the actions of another substance.

Association studies: Population-based genetic studies that examine whether an allele of a certain gene or marker co-occurs with a phenotype (e.g., a disease) at a significantly higher rate than predicted by chance alone.

Autonomic nervous system: Part of the nervous system whose primary function is to maintain homeostasis in the body; controls, for example, heart rate, digestion, respiration, and salivation. Most of its actions are involuntary.

Cannabinoids: Compounds naturally produced in the body that have a structure similar to the psychoactive compounds found in the cannabis plant and which serve as signaling molecules in the brain; the endogenous cannabinoid system is thought to regulate brain circuits using the neurotransmitter dopamine that likely play an important role in the rewarding experiences associated with addictive drugs.

Case–control study: Type of epidemiological study design used to identify factors that may contribute to a medical condition by comparing a group of patients who have that condition (e.g., alcohol dependence) with a group of patients who do not.

Cortisol: The most important and most potent glucocorticoid in humans; produced in the outer layer of the adrenal glands.

Corticosterone: The most important glucocorticoid in rodents; produced in the outer layer of the adrenal glands.

Dopamine: Neurotransmitter that is involved, among other functions, in controlling behavior and cognition, motor activity, motivation and reward, mood, attention, and learning. Dopamine-producing nerve cells are located primarily in a region of the midbrain called the ventral tegmental area and in regions of the hypothalamus.

Dopaminergic: Referring to nerve cells (i.e., neurons) that use dopamine as a neurotransmitter.

DNA (deoxyribonucleic acid): The molecule that carries the genetic code in all organisms except some viruses. DNA is composed of a sequence of nucleotides.

Endophenotype: A heritable trait or characteristic that is thought to be an intermediate phenotype between a genetic predisposition and a clinical disorder; for example, certain neurobiological characteristics have been noted in people with alcoholism and may be used as endophenotypes to identify people at risk for alcoholism. Endophenotypes are thought to be useful for gene identification under the assumption that they are simpler and closer to the genetic underpinnings of the disorder.

Epigenetic: Referring to heritable traits (over rounds of cell division and sometimes from one generation to the next) that do not involve changes to the underlying DNA sequence; epigenetic changes can alter the appearance and structure of the DNA and influence how a gene can interact with important regulatory factors.

Event-related potential: A characteristic pattern of brain waves elicited when a person is exposed to a sudden stimulus (e.g., a sudden sound or light).

Externalizing disorder: Psychiatric disorder that is characterized by disinhibited behavior, such as antisocial personality disorder, conduct disorder, or attention deficit/hyperactivity disorder.

Fear conditioning: An experimental strategy by which an animal or person learns to fear a new, neutral stimulus (e.g., a tone or being in a new environment); is achieved by pairing the neutral stimulus with an aversive stimulus (e.g., an electric shock or unpleasant smell) so that eventually the neutral stimulus alone can elicit a state of fear.

Gene–environment interaction: The modification of genetic risk factors by environmental risk and protective factors; alternatively, can be conceptualized as the role that specific genetic risk factors play in determining individual differences in vulnerability to environmental risk factors.

Gene mapping: Determination of the positions of genes on a chromosome relative to one another.

Genome: The total genetic material of an organism or species.

Genotype: The genetic makeup of an individual organism that is determined by the specific alleles of each gene carried by the individual. For genetically determined characteristics, differences in alleles among individuals account for the differences in phenotype observed among those individuals.

Glucocorticoids: A class of steroid hormones that, in addition to playing a central role in the stress response, are involved in the metabolism of the sugar glucose and maintenance of normal glucose concentrations in the blood, have anti-inflammatory effects, and can help suppress the immune response.

Glutamate: A neurotransmitter that enhances the activity of the signal-receiving nerve cell (i.e., an excitatory neurotransmitter); is involved in cognitive functions such as learning and memory; alcohol-induced increases in glutamate activity are probably involved in some manifestations of alcohol withdrawal.

Glutamatergic: Referring to nerve cells (i.e., neurons) that use glutamate as a neurotransmitter.

Haplotype: A set of closely linked genetic markers present on one chromosome that tend to be inherited together.

Heritability: Measure of the extent to which an observable characteristic (e.g., development of alcohol dependence) is determined by genetic (i.e., heritable) factors rather than by environmental factors.

Homeostasis: The maintenance of a stable, internal state or condition (e.g., body temperature or blood pressure) in a living organism.

Hypercortisolism: The presence of excessive levels of cortisol in the body caused mainly by abnormally high levels of adrenocorticoid hormone from the pituitary gland (e.g., because of a pituitary tumor); characterized by rapid weight gain and obesity, round face, excessive sweating, thinning of the skin that results in easy bruising, elevated blood pressure, and other signs and symptoms; also known as Cushing’s Syndrome.

Hypothalamic–pituitary–adrenal (HPA) axis: A hormone system involving the hypothalamus, pituitary gland, and adrenal glands that controls the stress response as well as regulates other body processes, such as digestion, the immune system, mood, and energy usage.

Hypothalamus: Brain region located deep inside the brain that links the nervous system and the hormone (i.e., endocrine) system; it regulates certain metabolic processes, controls functions such as body temperature, hunger, thirst, and fatigue; and produces hormones such as corticotrophin-releasing factor that in turn stimulate or inhibit the release of hormones from the pituitary gland.

Isozymes: Enzymes that differ in the sequence of their building blocks (i.e., amino acids) but that mediate the same chemical reaction.

Limbic system: A group of brain structures that together control such functions as emotion, behavior, and long-term memory by acting on the hormone (i.e., endocrine) system and the autonomic nervous system. Several structures are considered to be part of the limbic system, including the amygdala, hippocampus, hypothalamus, and thalamus. It also is connected with the nucleus accumbens.

Linkage analysis: A technique used to search for chromosomal regions likely to contain genes conferring risk for a disorder by testing for chromosomal regions where affected siblings are more likely to be sharing the same stretch of DNA.

Marker: A DNA sequence whose chromosomal location has been determined and which can be used in linkage analyses to try to identify genes; markers typically have multiple alleles.

Messenger RNA (mRNA): Key intermediary molecules that are generated when a gene is expressed (i.e., when the information encoded in the gene is converted into a protein product by the cell); mRNA levels for a gene are used as an indicator of how “active” the gene is (i.e., how much of the protein is produced).

Microdialysis: An experimental technique to determine the chemical composition of the fluid found in the spaces between cells (i.e., the extracellular space) in the tissues.

Negative affect: A negative emotion or subjectively experienced feeling, such as distress, anger, or fear.

Neuroactive steroids: Steroid hormones that act on the nervous system, altering the excitability of specific nerve cells.

Neurotransmitter: Signaling molecules produced in nerve signals that serve to transmit signals from one nerve cell to another nerve cell or from a nerve cell to another type of cell (e.g., muscle cell); neurotransmitters are released from the signal-emitting neuron and bind to receptors on the surface of the signal-receiving cell.

Nonsynonymous polymorphism: Polymorphism in which the different variants (i.e., alleles) result in the production of proteins with different amino acid sequences.

Nucleus (pl. nuclei): A structure in the central nervous system that is composed mainly of nerve cell bodies and which acts as a transit point for nerve signals in a specific neural subsystem.

Nucleus accumbens: A nucleus located in the forebrain that plays an important role in reward, pleasure, addiction, and fear; primarily contains nerve cells that produce the neurotransmitter γ-aminobutyric acid.

Opioids: Small molecules naturally produced in the body that have an effect similar to that of the opiates (e.g., morphine and heroin) and which, among other functions, modulate the actions of other neurotransmitters.

Peptide: A small molecule composed of a short chain of the building blocks of proteins (i.e., amino acids); in contrast, proteins are defined as long chains of amino acids.

Phenotype: The observable structural or functional characteristics of an individual organism that result from the interaction of its genotype with environmental factors.

Pituitary gland: A small gland located at the base of the brain, just below the hypothalamus; releasing factors produced by the hypothalamus stimulate the pituitary gland to secrete a variety of hormones that in turn regulate homeostasis, thereby controlling growth, blood pressure, sex organ function, and other functions; also part of the hypothalamic– pituitary–adrenal axis.

Polymorphism: The presence of two or more alleles of a gene or other DNA sequence in a population.

Positron emission tomography (PET): An imaging technique that produces a three-dimensional image of functional processes in the body; involves the introduction of a radioactively labeled, metabolically active molecule whose activity can be detected using specific scanners; the distribution of the signals detected is used to generate a three-dimensional image of the respective structures.

Pseudo-Cushing Syndrome: Medical condition in which patients exhibit the signs and symptoms of hypercortisolism but which is not caused by a problem with the hypothalamic–pituitary–adrenal axis.

Receptor: A protein molecule located in the membrane surrounding a cell or in the cell’s interior that can bind with a signaling molecule (e.g., a hormone or neurotransmitter), thereby setting off a chain of biochemical reactions in the cell that alter the cell’s behavior in a specific manner.

Salience: A state or property of an item or a stimulus that makes it stand out from other items or experiences. People normally focus their attention on the stimuli with the greatest salience; however, what is considered salient is often determined by emotional or motivational factors and not necessarily by physical factors (e.g., intensity or size of a stimulus).

Single-nucleotide polymorphism (SNP): A variation in the DNA sequence of the genome that occurs when a single DNA building block (i.e., nucleotide) differs between members of a species.

Striatum: A part of the brain that is involved in the planning of movement as well as in other cognitive processes; in humans the striatum is activated by stimuli associated with reward but also with aversive, novel, unexpected, or particularly intense stimuli with high salience; includes several nuclei, including the caudate nucleus, putamen, and nucleus accumbens.

Sympathetic nervous system: A branch of the autonomous nervous system that becomes more active at times of stress; its actions during the stress response comprise the fight-or-flight response.

Synapse: The site where two neurons interact and transmit signals from one neuron to the next.

Transcription factor: A protein that regulates gene expression (i.e., the conversion of the information encoded in a gene into a protein product) by binding to specific sites on the DNA, thereby activating other proteins involved in the conversion process.

Twin study: Type of behavioral genetic study that compares the similarity between identical (i.e., monozygotic) twins, who share 100 percent of their genes, to that of fraternal (i.e., dizygotic) twins, who share only 50 percent of their genes, in order to determine the contribution of genetic versus environmental factors on a given characteristic. If the similarity is found to be greater among monozygotic than dizygotic twins, genetic factors play a role in the characteristic under investigation.

